# Active inference and the two-step task

**DOI:** 10.1038/s41598-022-21766-4

**Published:** 2022-10-21

**Authors:** Sam Gijsen, Miro Grundei, Felix Blankenburg

**Affiliations:** 1 Neurocomputation and Neuroimaging Unit, Freie Universität Berlin, 14195 Berlin, Germany; 2 Berlin School of Mind and Brain, Humboldt-Universität zu Berlin, 10117 Berlin, Germany

**Keywords:** Human behaviour, Cognitive neuroscience, Computational neuroscience

## Abstract

Sequential decision problems distill important challenges frequently faced by humans. Through repeated interactions with an uncertain world, unknown statistics need to be learned while balancing exploration and exploitation. Reinforcement learning is a prominent method for modeling such behaviour, with a prevalent application being the two-step task. However, recent studies indicate that the standard reinforcement learning model sometimes describes features of human task behaviour inaccurately and incompletely. We investigated whether active inference, a framework proposing a trade-off to the exploration-exploitation dilemma, could better describe human behaviour. Therefore, we re-analysed four publicly available datasets of the two-step task, performed Bayesian model selection, and compared behavioural model predictions. Two datasets, which revealed more model-based inference and behaviour indicative of directed exploration, were better described by active inference, while the models scored similarly for the remaining datasets. Learning using probability distributions appears to contribute to the improved model fits. Further, approximately half of all participants showed sensitivity to information gain as formulated under active inference, although behavioural exploration effects were not fully captured. These results contribute to the empirical validation of active inference as a model of human behaviour and the study of alternative models for the influential two-step task.

## Introduction

Sequential decision problems capture an important essence of the challenges regularly encountered by humans. Although the environmental structure and statistics often cannot be observed directly, they may be inferred through repeated interactions. Especially in case of a dynamically changing environment, a host of strategies have been proposed to underlie human decision making. Reinforcement learning is eminently cast as pursuing the long-term maximization of scalar reward^1^. This approach can either be *model-free* or *model-based*. A model-free approach states that action-selection proceeds based on the extent to which an action has been reinforced in the past. However, such a strategy ignores available knowledge about the environmental structure and is difficult to reconcile with goal-directed actions, consequently failing to capture important aspects of human behaviour^2^. In contrast, a model-based strategy is able to exploit structural knowledge in pursuit of goals and is therefore capable of predicting action outcomes.

The two-step task provides a prominent example of the application of reinforcement learning to the study of human decision-making^3^. The task requires the sequential traversal of two stages via binary action selection to accumulate rewards or avoid punishment. Specifically, the task was designed to disambiguate between model-free and model-based strategies. Model-based inference uses the probabilistic transition between the stages to steer itself towards lucrative states, while a model-free approach foregoes such transition-based planning and instead only relies on observed stimulus-action mapping. The two-step task has been highly influential and has seen widespread adoption, including the study of pathology such as obsessive-compulsive disorder^4^ and gambling disorder^5^. This research has generally used the hybrid reinforcement learning model as introduced by Daw et al.^3^, which combines independent model-free and model-based strategies. However, it has been shown that not all aspects of human behaviour on the two-step task are captured by this commonly-used reinforcement learning model^6^. Furthermore, it has been argued that the hybrid model may mischaracterise model-free behaviour as model-based^7^, or vice versa^6,8^.

Sequential decision problems additionally invoke the exploration-exploitation trade-off. Do we choose an option that is well-understood and known to be rewarding? Or should we risk foregoing this immediate reward so as to learn more about alternatives and in doing so potentially find an even more rewarding option? This conflict has been a prominent area of research in psychology^9^, neuroscience^10,11^, and computer science^1,12^. Exploration behaviour can result from stochasticity in action selection, randomizing choice rather than deterministically choosing the most rewarding action (random exploration). Additionally, action-values may not be purely reward-based but can receive an additional information bonus, biasing actions toward uncertain options (directed exploration). Directed exploration therefore describes an intentional process to minimize information discrepancies between options, which has so far only been observed in a subset of studies on human behaviour^11,13–15^. It has been a particularly powerful descriptor of behaviour in the domain of visual sensing, in which efficient exploration is likely the primary goal^16–18^. However, as directed exploration is not part of the hybrid reinforcement learning model, its potential role in the two-step task has not received much attention. Given that the two-step task features outcome-probabilities that drift over time, a model-based approach may benefit from an information gathering mechanism to promote exploration and discover rewarding actions. This begs the question whether further variance in behavioural data may be explained by modeling directed exploration dynamics.

Active inference has been proposed in neuroscience as a framework for describing the exploration-exploitation trade-off^19^. Derived from the free energy principle^20^, active inference leverages the concept of a generative model that is iteratively optimized and allows for planning and decision making^21^. By taking a probabilistic inference approach, it is closely related to the Bayesian-brain hypothesis^22,23^. Action selection is cast as a minimization of *expected free energy*, which combines terms for both the realization of an agent’s preferences and exploration. Under active inference, exploration results from seeking information gain, for which the generative model is used to infer the degree to which actions and their resulting observations may change belief distributions. Such a choice bias promotes directed exploration until the agent gains confidence in its understanding of its environment. At that point, further observations have a diminished impact on beliefs and the realization of preferences becomes a more dominant determinant of behaviour.

Active inference has a body of theoretical work^24,25^ and has been studied in-silico^26–28^. However, empirical validation based on human-behaviour has only recently started to emerge. For example, active inference was used to characterise atypical choice behaviour in individuals with substance use disorder^29,30^ and various other psychopathology^31,32^. Furthermore, model parameters fitted to human behaviour have been shown to correlate across time^33^ and to potentially hold predictive power of future symptomatology^30^. Congruent with active inference predictions, human choice behaviour has also been shown to not merely be a function of reward or utility, but also entropy maximization^34,35^. Nevertheless, it is unclear whether a central feature of the framework, namely its proposed resolution to the exploration-exploitation dilemma, is able to capture human behaviour better than existing models in a variety of task settings. This is an important aspect of the framework, given that without the information-gain incentive, especially on simpler tasks, active inference can reduce to generate highly similar behaviour as a purely reward-maximizing reinforcement learning agent.

In the current study, we leverage the widely studied two-step task to investigate the suitability of active inference as a description of human choice behaviour. To this end, we use four publicly available datasets. We compare the behavioural predictions of active inference to those of the hybrid reinforcement learning model and perform Bayesian model selection analyses. By doing so, we contribute to the emerging, empirical study of active inference as well as investigate an alternative model for behaviour on the two-step task.

## Methods

### Participants and behavioural task

**Figure 1:**
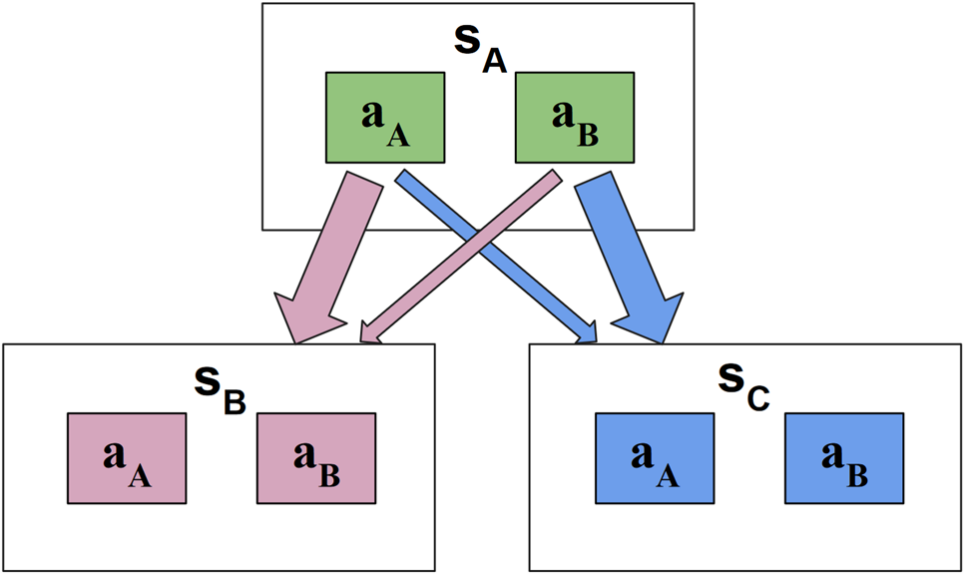
A graphical abstraction of the two-step task. Each trial always starts in the same (here, green) initial state $s_A$ where participants choose between two green options. Each option is associated with a common and rare transition to one of two final-stage states. The green option $a_A$ here has a 0.7 probability to move the participant to the pink $s_B$ final state (common transition), and a 0.3 probability to transition to the blue $s_C$ final stage (rare transition). The transition probabilities for the green option $a_B$ are the opposite of those of $a_A$. Both final-stage states have two further options to choose from (pink $a_A$ vs. $a_B$ and blue $a_A$ vs. $a_B$), which are associated with a binary outcome probability, each drifting independently over time The exact stimuli vary between studies.

We studied human behavioural data in the two-step task originally designed by Daw et al.^3^. In this paradigm, participants first choose from two available initial-stage actions, each of which is uniquely associated with a likely (*p* = 0.7) and unlikely (*p* = 0.3) state transition to one of two final-stage states. There, another decision needs to be made between two final-stage actions, which yields a binary outcome. The outcome probability for each second-stage action follows an independent Gaussian random walk (Fig. 1).

The two-step task was initially designed to disambiguate between model-free and model-based strategies. A pure model-free strategy solely relies on learning from observed stimulus-action mapping. In contrast, model-based reasoning may use the known latent structure of transitions between initial-stage actions and final-stage states. The two-step task allows for the distinction between these two strategies by means of model comparison as well as analyses of averaged responses. The latter relies on the insight that model-free inference will lead to a greater probability to repeat an initial-stage action if it lead to the preferred outcome on the previous trial, independent of transition type. Model-based inference, in contrast, exploits the knowledge of state transitions and tends to repeat this initial-stage action only following a common transition. In case of a rare transition, the agent becomes more likely to switch to the other initial-stage action so as to increase its probability to access the promising, final-stage action.

The data was obtained from four publicly available datasets. These datasets were selected due to their similar task structure. First, 197 subjects participated in the online ’Daw two-step task’ by Kool et al.^36^. Two further datasets were made available by da Silva and Hare^6^. These “Magic Carpet” (n = 24) and “Spaceship” (n = 21) experiments focused on providing intuitive and thorough instructions regarding all aspects of the task. Finally, the “Shock” dataset by Lockwood et al.^37^ consists of 36 participants and differs from the aforementioned three experiments by using future electric shocks (or their absence) as the binary outcome rather than a monetary reward (or its absence). In addition, this study features two conditions in which participants are told the electric shocks will either be delivered to themselves (’self’ condition) or another, anonymous participant (’other’ condition). Finally, it used different parameters for the Gaussian random walk ($\mu =0$, $\sigma =0.2$, reflecting boundaries at [0, 1]) than the other datasets ($\mu =0$, $\sigma =0.025$, reflecting boundaries at [0.25, 0.75]). All participants were explicitly told about the task structure and received training on the task prior to data-collection. Please refer to the original manuscripts for full experimental descriptions.

### Logistic regression analyses

Miller et al.^38^ introduced and verified a logistic regression analysis of initial-stage choice behaviour based on behaviour, transitions, and outcomes on multiple preceding trials in the two-step task. The method thereby allows for insight into how the local history of these factors influences decision making without relying on a computational model. Here we adopt this analysis to describe behaviour across datasets and to validate model predictions of behaviour. The analysis was shown to alleviate issues of the original regression analyses introduced by Daw et al.^3,38^, which only considered the previous trial, by providing more accurate descriptions of behaviour and being less sensitive to learning rates. da Silva and Hare^6^ used a variation of this method to describe aspects of behaviour that are not visible in case only the previous trial is considered and showed the shortcoming of the hybrid reinforcement learning model in capturing this behaviour. Specifically, da Silva and Hare^6^ model the final-stage outcome in trial *t* as $o_t=+1$ for positive outcomes (monetary rewards or absence of shocks) and $o_t=-1$ otherwise (shocks or absence of monetary rewards), while transitions are coded as $\tau _t=+1$ for common transitions and $\tau _t=-1$ for rare transitions. The dependent variable *y* is the initial-stage action (i.e., which of the two stimuli was chosen) on trial $t'$, coded as $-1$ and +1. Then, for each trial *t* of the $T=4$ preceding trials, $x_t$ denotes the initial-stage choice made on that trial. This yields the following regression model:

1 $$\begin{aligned} \log \left( \frac{p(y_t=+1)}{p(y_t=-1)} \right) = \sum _{t=1}^T \beta _0^{t'-t} x_{t'-t} + \beta _o^{t'-t} o_{t'-t} x_{t'-t} + \beta _\tau ^{t'-t} \tau _{t'-t} x_{t'-t} + \beta _{o \times \tau }^{t'-t} o_{t'-t} \tau _{t'-t} x_{t'-t} \end{aligned}$$

where the $\beta$-coefficients indicate the influence of variables in trial $t'-t$ on the initial-stage choice in trial $t'$: $\beta _0^{t'-t}$ quantifies the influence of the initial-stage choice, $\beta _o^{t'-t}$ quantifies the effect of observation, $\beta _\tau ^{t'-t}$ quantifies the effect of transition, and $\beta _{o \times \tau }^{t'-t}$ quantifies the interaction between observation and interaction. The model was fit for each participant separately using Scikit-learn^39^. To apply the analysis to the computational models and mitigate the randomness of choice selection, each participant’s maximum likelihood parameters were used to simulate model behaviour 20 times. The resulting regression coefficients were averaged across iterations.

### Computational modeling

In order to investigate the role of directed exploration on the two-step task, two families of models were implemented. A description of transition probability learning is followed by the common reinforcement learning approach and by a probabilistic framework implementing active inference with information-gain incentives. Parameters of both models are fit to participant behaviour and relative model performance is analysed via Bayesian model comparison. The initial-stage had only one state $s_A$ (green in Fig. 1), while the final-stage had two possible states: $s_B$ and $s_C$ (pink and blue in Fig. 1). Each state had two actions, denoted $a_A$ and $a_B$. On trial *t*, the initial-stage state is denoted by $s_{1,t}$ and the final-stage state $s_{2,t}$, and similarly actions by $a_{1,t}$ in the initial-stage and $a_{2,t}$ in the final-stage. Outcomes $o_t$ refer to the binary, final-stage observations, consisting of the absence or presence of a monetary reward or a future electric shock depending on the dataset. As these only occur in the final-stage, they are always zero in the initial-stage.

#### Transition learning

The transition structure $p(s_{2,t} \vert s_{1,t}, a_{1,t})$ specifies how the two available initial-stage actions may transition the agent from $s_{1,t}$ to $s_{2,t}$. As each dataset included a familiarization and training phase prior to the start of the experiment, participants were aware that the transition probabilities of initial-stage actions were mirrored and could either be $p(s_B \vert s_A, a_A)=p(s_C \vert s_A, a_B)=0.7$ or $p(s_B \vert s_A, a_A)=p(s_C \vert s_A, a_B)=0.3$ (with $p(s_B \vert s_A, a_A)=1-p(s_C \vert s_A, a_A)$ and $p(s_B \vert s_A, a_B)=1-p(s_C \vert s_A, a_B)$). Consequently, which initial-stage action commonly led to which final-stage state was unknown at the start of the task and thus had to be inferred. Here, we model this transition learning similar to previous studies, by having agents count transitions and on each trial choose the most likely transition structure based on the observed frequencies. The three options included the two possible true structures described above as well as flat (*p* = 0.5) transition probabilities^3,6,36^. This simple strategy settles on the correct solution after only a few trials and allows for the use of identical transition learning between the active inference and model-based reinforcement learning models. More sophisticated methods would have a limited contribution as the correct solution is found quickly and does not change across the experiment. As the identity of the states could be directly observed in all studies, the agents also have access to this information without the need for inference.

#### Hybrid reinforcement learning

The hybrid reinforcement learning model combines the action evaluations of model-free and model-based algorithms. The model was introduced by Daw et al.^3^, who used it to quantify the relative contributions of these two strategies to behaviour. Both strategies map state-action pairs to their expected discounted future return, captured by *Q*(*s*, *a*). The model-free strategy corresponds to the State-action-reward-state-action (SARSA($\lambda$)) algorithm^40^, which updates the value of state-action pairs *s*, *a* at stage $p=\{1,2\}$ as follows:

2 $$\begin{aligned} Q_{MF}(s,a)&= Q_{MF}(s,a) + \alpha _p \delta _{p,t} \lambda _{p,t} (s,a) \end{aligned}$$

3 $$\begin{aligned} \delta _{p,t}&= o_t + Q_{MF} \left( s_{p+1,t}, a_{p+1,t} \right) - Q_{MF} (s_{p,t}, a_{p,t}) \end{aligned}$$

where $\delta$ is the outcome prediction error, $\alpha$ ($\alpha _1$ or $\alpha _2$ depending on the stage) is the learning rate, and $\lambda$ is the eligibility parameter, modulating the effect of the final-stage prediction error on the values of initial-stage actions. Due to there not being an outcome on the initial-stage, the prediction error for this stage depends on the value of the selected final-stage action. The final-stage prediction error depends on the outcome $o_t$ and thus the $Q-$values for both stages are updated at the final stage, using the following errors:

4 $$\begin{aligned} \delta _{1,t}&= Q_{MF}(s_{2,t},a_{2,t}) - Q_{MF}(s_{1,t}, a_{1,t}) \end{aligned}$$

5 $$\begin{aligned} \delta _{2,t}&= o_t - Q_{MF}(s_{2,t}, a_{2,t}) \end{aligned}$$

The model-based algorithm differs from the model-free approach by using knowledge about the transitions between initial-stage actions and final-stage states. Final-stage actions are evaluated directly from prediction errors as in model-free learning, however, the value of each initial-stage action $a_j$ depends on its probabilistic mapping to final-stage states (and thereby to final-stage actions).

6 $$\begin{aligned} Q_{MB}(s_A, a_j) = p(s_B|s_A, a_j) \max _{a_2 \in {\mathcal {A}}_B} Q_{MF}(s_B,a_2) + p(s_C|s_A, a_j) \max _{a_2 \in {\mathcal {A}}_C} Q_{MF}(s_C,a_2) \end{aligned}$$

where ${\mathcal {A}}_B$ and ${\mathcal {A}}_C$ are the sets of available actions in the respective final-stage states ($s_B$ and $s_C$ respectively).

The Q-values of the model-free and model-based algorithms are combined according to the weighting parameter *w*:

7 $$\begin{aligned} Q_{net}(s_A,a_j) = w Q_{MB}(s_A, a_j) + (1-w) Q_{MF} (s_A, a_j) \end{aligned}$$

Note that there is no need to weigh model-free and model-based estimates for final-stage choices as the algorithms do not differ there. Furthermore, the hybrid model includes pure model-free and model-based inference as special cases for $w=0$ and $w=1$, respectively. Next, an action is selected using a softmax operator:

8 $$\begin{aligned} p(a_{p,t} = a|s_{p,t}) = \frac{\exp (\beta _p Q_{net}(s_{p,t},a) + \rho \times rep(a) )}{\sum _{a'} \exp (\beta _p Q_{net} (s_{p,t},a') + \rho \times rep(a') )} \end{aligned}$$

where $\rho$ is a commonly included parameter modeling initial-stage response stickiness and $rep(a')$ is 1 if *a* is the initial-stage action that was chosen in the last trial and 0 otherwise^36^, and $\beta$ is the inverse temperature parameter that controls the randomness of the action selection. Separate $\beta _1$ and $\beta _2$ parameters are fitted for each stage to allow for different levels of choice randomness.

#### Active inference

Active inference agents rely on a generative model of the task. The estimated transition probabilities (denoted by $\theta _1$) and outcome probabilities ($\theta _2$; please see below) are together denoted as $\theta$, with the generative model taking the following form:

9 $$\begin{aligned} { p(o_t, s_{2,t} \vert s_{1,t} , \theta ) = p(o_t| s_{2,t} , \theta ) p(s_{2,t} \vert s_{1,t}, \theta ) p(\theta ) } \end{aligned}$$

In the current study we focus on information-gain incentives of final-stage outcome probabilities $\theta _2$ and omit state inference incentives from active inference due to the static transition probabilities $\theta _1$ and observable state identity. Note that if it is assumed for participants following training to be aware that the transition structure is one of two mirrored options (*p* = [0.3 0.7] or *p* = [0.7 0.3]), then both initial-stage actions provide equal amounts of information about transition probabilities. As a result, action-selection will only be sensitive to information discrepancies about outcome probabilities.

Under active inference, agents resolve the exploration-exploitation dilemma by basing action selection on a single expression. Actions are more probable to be selected if they minimise expected surprise about future observations^21^, that is, if they minimise expected free energy. This quantity can be expressed in variety of ways, with an intuitive decomposition featuring extrinsic and intrinsic value terms^21^:

10 $$\begin{aligned} G_t(a) = - \underbrace{E_{p(o_t;\pi _t(\theta _2) \vert a_t=a)} \left[ \ln p(o_t|C) \right] }_\text {Extrinsic Value} - \underbrace{E_{p(o_t;\pi _t(\theta _2) \vert a_t=a)}\left[ D_{KL} \left( \pi _t(\theta _2) \vert o_t, a_t=a \Vert \pi _t(\theta _2) \right) \right] }_\text {Intrinsic Value} \end{aligned}$$

where $p(o_t \vert C)$ denote the prior preferences over outcomes and $D_{KL}$ is the Kullback-Leibler divergence, here between beliefs $\pi _t (\theta _2)$ about final-stage outcome probabilities $\theta _2$ before (prior) and after (posterior) hypothetically observing an outcome resulting from action selection. $p(o_t; \pi _t(\theta _2) \vert a)$ can be understood as the distribution obtained from $p(o_t \vert \theta _2, a) \pi _t(\theta _2)$ once $\theta _2$ has been marginalised out, noting that $\pi _t(\theta _2 \vert a) = \pi _t(\theta _2)$ by construction. For a given action, the extrinsic value term is a measure of how likely prior preferences are to be attained, while the intrinsic value term quantifies the expected information gain. The formulation above suffices for final-stage action selection, however, for the initial-stage the action-dependent state transitions need to be accounted for. As state inference was not included, this leads to the simplified setting wherein both prior preference realization and information-gain is limited to the final-stage actions. For a multi-step policy, we formulate the computation of the expected free energy for an initial-stage action $a_j$. $G(a_j)$ depends on the probabilistic mapping of the action to final-stage states $s_2$ and, by extension, the final-stage actions that are available in these states. Specifically, the estimated action-specific transition probabilities are multiplied by the expected free energy of the associated final-stage actions:

11 $$\begin{aligned} {G(a_j) = p(s_B | s_A,a_j,\theta _1) \sum _{a_2 \in {\mathcal {A}}_B} G(a_2) + p(s_C|s_A,a_j,\theta _1) \sum _{a_2 \in {\mathcal {A}}_C} G(a_2)} \end{aligned}$$

where ${\mathcal {A}}_B$ and ${\mathcal {A}}_C$ are the sets of available actions in the corresponding final-stage states ($s_B$ and $s_C$ respectively).

The prior preferences capture the relative attractiveness of the different outcomes, with desired outcomes being assigned higher probabilities. Here we constrain the prior preferences regarding action outcomes to a Bernoulli distribution implying $o_t=1$ is preferred over $o_t=0$ following Marković et al.^27^:

12 $$\begin{aligned} P(o_t \vert C) = \frac{1}{Z(\lambda )} e^{o_t \lambda } e^{-(1-o_t)\lambda }. \end{aligned}$$

The $\lambda$-parameter specifies the precision of the prior preferences. For $\lambda =0$ (i.e., zero precision), the outcomes are valued equally and the agent will thus only maximize intrinsic value, corresponding to pure information gathering about the outcome probabilities encoded by $\pi _t(\theta _2)$. As $\lambda$ increases, the agent will value information-gain less and focus more on realizing prior preferences, thereby becoming increasingly risk-seeking. This precision parameter thus balances exploratory and exploitative behaviour.

Previous studies have modeled a tendency for participants to repeat initial-stage actions independent of the outcome^36^. Such behaviour may be modelled under active inference as a habit, which we here assume to be static across the experiment for simplicity, again constrained to a Bernoulli distribution:

13 $$\begin{aligned} E_a (a_j)&= \frac{1}{Z(\kappa )} e^{\delta _{a_{t-1},a_j} \kappa } e^{-(1-\delta _{a_{t-1},a_j}) \kappa } \end{aligned}$$

with precision parameter $\kappa$ and $E_a(a)$ always set to zero for final-stage actions. Action selection at stage $p = \{1,2\}$ may then proceed by applying a softmax operation ($\sigma$) to the expected free energies, together with the habitual bias:

14 $$\begin{aligned} p(a_{p,t}) = \sigma \left[ -\gamma _p G(a_{p,t}) + E_a \right] \end{aligned}$$

with $\gamma _p$ functioning as an inverse temperature parameter, controlling the stochasticity of action selection. As in the hybrid model, two separate $\gamma$ parameters are fit to participant data, allowing for different levels of randomness in initial- and final-stage action selection.

Finally, previous studies on multi-armed bandit tasks have found evidence for a bias in prior outcome probabilities^41^. To capture any such biases and their effects on action selection, the mean ($E \left[ \pi _0(\theta _2) \right] = \frac{\alpha _0}{\alpha _0+\beta _0}$) of the prior Beta distribution is included as a free parameter in the model fitting procedure (please see below).

Next, we describe the learning rule of observations for the active inference models. This necessarily deviates from the hybrid reinforcement learning model as the aforementioned computations require probability distributions, rather than point estimates. Note that this only concerns the learning of final-stage outcome probabilities, as transition learning is shared across all models as detailed above. Liakoni et al.^42^ introduced surprise-based learning algorithms for changepoint paradigms that feature occasional resampling of the environmental statistics. We will briefly describe one such algorithm and subsequently modify it to better suit the current environment with drifting parameters.

For brevity, references to *s* and *a* have their subscripts dropped as they will generally refer to the final-stage. Wherever the initial-stage state or actions are concerned, these will be explicitly denoted by $s_1$ and $a_1$. This process of observation emission corresponds to sampling from a Bernoulli distribution parameterized by an expectation $\theta _{2,a}$ for each final-stage action, encoding the probability of observing $o_t = 0$ or $o_t=1$. A Bayesian agent requires a prior distribution over the estimated $\theta _2$ probabilities, for which conjugate Beta priors are appropriate:

15 $$\begin{aligned} p(\theta _2)&= \prod _{s=1}^2 \prod _{a=1}^2 {\mathcal {B}}e (\alpha _{s,a}, \beta _{s,a}) \end{aligned}$$

In such a setting, Bayesian inference corresponds to the following simple update rules for the parameters of the Beta distributions:

16 $$\begin{aligned} \alpha _{s,a}&= \alpha _{s,a} + \delta _{a_t,a} o_t \end{aligned}$$

17 $$\begin{aligned} \beta _{s,a}&= \beta _{s,a} + \delta _{a_t,a} (1-o_t) \end{aligned}$$

with $\delta$ being the Kronecker delta, which is 1 if both variables are equal, and 0 otherwise. However, such updating will quickly lead to inflexible beliefs, making it unsuited for dynamic environments. Liakoni et al.^42^ propose an updating scheme based on the Bayes-factor surprise $S_{BF} {\ge 0}$, a ratio between the subjective probability of an observation under the current beliefs and prior beliefs. Together with a prior belief of the volatility of the environment, $\nu {\in [0,1]}$, this surprise quantity enables a simple learning rule that moves the current belief distribution over final-stage outcome probabilities $\pi _t(\theta _2)$ closer to the uninformed prior beliefs $\pi _0(\theta _2)$ (here: ${\mathcal {B}}e(\alpha _0, \beta _0)$) based on the degree to which current observations are more likely under these prior parameters. This is achieved by scaling concentration parameters with a surprise-modulated adaptation rate $\chi {\in [0,1]}$. The rate for the current trial $\chi _t$ may be computed as follows:

18 $$\begin{aligned} \chi _t&= {\chi }(S_{BF},m) \end{aligned}$$

19 $$\begin{aligned} \chi (S,m)&= \frac{mS}{1+mS} \end{aligned}$$

20 $$\begin{aligned} S_{BF} &= \frac{p(o_t; \pi _0(\theta _2))}{ p(o_t; \pi _t(\theta _2))} \\ m &= \frac{\nu }{1-\nu } \end{aligned}$$

21 $$\begin{aligned} p \left( o_t; \pi _t(\theta _2) \right)&= {\left\{ \begin{array}{ll} \frac{\alpha _{s,a,t}}{\alpha _{s,a,t} + \beta _{s,a,t}} \text { if } o_t = 0 \\ \frac{\beta _{s,a,t}}{\alpha _{s,a,t} + \beta _{s,a,t}} \text { if } o_t = 1 \end{array}\right. } \end{aligned}$$

where $m \ge 0$ depends on volatility *v* and modulates the effect of surprise on learning and $p(o_t; \pi (\theta _2))$ refers to the subjective probability of observing $o_t$ under the belief $\pi (\theta _2)$, which is easily computed using the Beta-parameters $\alpha$ and $\beta$. Liakoni et al.^42^ continue to derive the following update rules for the Beta-distributions:

22 $$\begin{aligned} \alpha _{s,a,t}&= (1-\chi _t)\alpha _{s,a,t-1} + \chi _t \alpha _0 + \delta _{a_t,a} o_t \end{aligned}$$

23 $$\begin{aligned} \beta _{s,a,t}&= (1-\chi _t) \beta _{s,a,t-1} + \chi _t \beta _0 + \delta _{a_t,a}(1-o_t) \end{aligned}$$

Effectively, surprising events shrink the concentration parameters of $\pi _t(\theta _2)$ towards those of $\pi _0(\theta _2)$, causing previous observations to be forgotten and thereby increasing the effect current observations have on the belief distribution, enabling flexible learning. In the current drift paradigm we are not interested in weighing between a changepoint or continuation, but rather the degree to which the generative probability has diffused and is thereby incompatible with currently held beliefs. This discrepancy between beliefs and the world can be quantified by predictive surprise ($PS(o_t) {\ge 0}$), which has a significant relevance in behavioural and imaging neuroscience^43–46^ as well as active inference^19^. The influence of surprise on beliefs may then be mediated by a prior volatility parameter $\nu _{PS} {\in [0,1]}$:

24 $$\begin{aligned} \chi _t&= {\chi }(PS,m) \end{aligned}$$

25 $$\begin{aligned} \chi (S,m)&= \frac{mS}{1+mS} \end{aligned}$$

26 $$\begin{aligned} PS&= -\ln p(o_t;\pi _t(\theta _2)) \end{aligned}$$

27 $$\begin{aligned} m&= \frac{\nu _{PS}}{1-\nu _{PS}} \end{aligned}$$

The update rules for the parameters corresponding to sampled actions then remain similar:

28 $$\begin{aligned} \alpha _{s,a,t}&= (1-\chi _t)\alpha _{s,a,t-1} + \delta _{a_t,a} o_t l \end{aligned}$$

29 $$\begin{aligned} \beta _{s,a,t}&= (1-\chi _t) \beta _{s,a,t-1} + \delta _{a_t,a}(1-o_t) l \end{aligned}$$

where *l* indicates a learning rate that may be fitted per participant and thus does not have to equate to 1. As probabilistic learning models are understudied in the context of the current paradigm, we also consider the possibility that beliefs decay independent of observations, akin to static forgetting. This may apply to beliefs of sampled actions, instead of or in addition to surprise-based learning, and unsampled actions, where it signals a gradual loss of confidence in beliefs about unexplored options, independently of $PS(o_t)$:

30 $$\begin{aligned} \alpha _{s,a,t}&= (1-\nu )\alpha _{s,a,t-1} + \nu \alpha _0 \end{aligned}$$

31 $$\begin{aligned} \beta _{s,a,t}&= (1-\nu )\beta _{s,a,t-1} + \nu \beta _0 \end{aligned}$$

where $\nu$ indicates a prior volatility parameter, with separate parameters $\nu =\{ \nu _{SD}, \nu _{UD} \}$ for sampled and unsampled actions respectively. Here, beliefs are assumed to decay back to the prior distributions over time rather than shrinking to zero, as very small concentration parameters may lead to computational instability.

**Figure 2:**
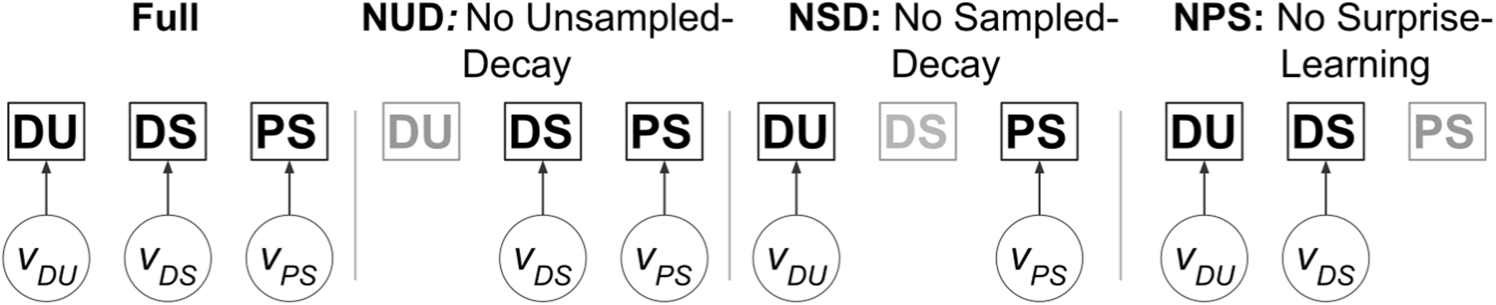
Graphic summary of learning models. Four variants of the learning model are compared, differentiated by their forgetting kinetics with associated prior volatility parameters $\nu$. The NUD and NSD models omit decay of concentration parameters of beliefs for unsampled and sampled actions respectively (corresponding to $\nu _{UD}=0$ and $\nu _{SD}=0$), while the NPS model excludes surprise-based learning ($\nu _{PS}=0$). The more complex Full model incorporates all three learning dynamics.

We formulate variations of the learning model that allow for surprise-based learning, static forgetting implemented as decay of concentration parameters, or both (Fig. 2). The rates of these mechanisms are governed by free volatility parameters. The resulting model-based hypotheses may be compared via model-comparison. Many further model variants may be hypothesized, including the possibility of ’shared’ parameters between forgetting and surprise-learning. We limit the analysis to a smaller amount of models for two reasons. First, the binary reward structure of the task was deemed likely to provide insufficiently detailed behavioural data to dissociate large numbers of highly similar models. Second, the current work aims to understand the explanatory power of active inference in the two-step task, rather than an exhaustive study of underlying learning dynamics. A comparison between potential key features of learning (decay and surprise-based learning) was performed to approximate an adequate level of flexibility to enable inference on the action-selection level. Hereby we intend to ameliorate the impact of potential complex interactions between learning dynamics and action-selection on model fitting.

#### Model fitting and model comparison

In order to fit the free parameters of the computational models to the participant choice data we used a constrained minimization algorithm (’L-BFGS-B’ as implemented in Scipy^47^). To mediate the problem of local optima, the optimization was ran 25 times for each participant with different (uniformly) randomized initializations for all parameters. The iteration that yielded the highest log likelihood was used for the model comparison procedures.

Fixed-effects model comparisons were performed using the Akaike’s Information Critria (AIC) and Bayesian Information Criteria (BIC).

32 $$\begin{aligned} BIC&:= k \ln (n) - 2 \ln ({\hat{L}}) \end{aligned}$$

33 $$\begin{aligned} AIC&:= 2k - 2 \ln ({\hat{L}}) \end{aligned}$$

with *k* being the number of free parameters in the model, *n* the amount of trials, and ${\hat{L}}$ denoting the maximized value of the subject- and model-specific log likelihood function. These approximations of log model evidence were then subjected to random-effects Bayesian model selection as implemented in SPM ($spm\_BMS.m$; Wellcome Trust Centre for Neuroimaging, Institute for Neurology, University College London, London, UK). This algorithm yields model-specific exceedance probabilities, representing the probability that the model has a higher frequency than the other included models on the group-level. The method additionally provides protected exceedance probabilities, which are more conservative by accounting for the possibility that apparent differences in model frequencies arise due to chance^48^. Models that share certain characteristics may be grouped into ’families’, and the subsequent comparison of families rather than individual models allows for inference on the contribution of these characteristics to the model fit^49^. This analysis is implemented in SPM’s $spm\_compare\_families.m$, for which we set priors to be equal across families rather than models (“F-unity” priors). As protected exceedance probabilities are not available for family-level inference, we present exceedance probabilities for these analyses.

To investigate the strengths and shortcomings of models in their ability to describe participant data, the models were used to simulate behaviour on the two-step task. The subject-specific parameters that maximized the log likelihood, as described above, were used to simulate action selection on the two-step task for 20 independent runs. Identical logistic regression analyses were then applied to this synthetic choice data, of which the resulting beta-coefficients allow for a comparison to the participant-derived coefficients. For $\lambda$-parameter reliability analyses, these synthethic datasets were subsequently subjected to the parameter-fitting procedure. The recovered $\lambda$-parameters were then compared with the parameter values fitted to the subject’s actual choice data.

## Results

### Logistic regression analyses

**Figure 3:**
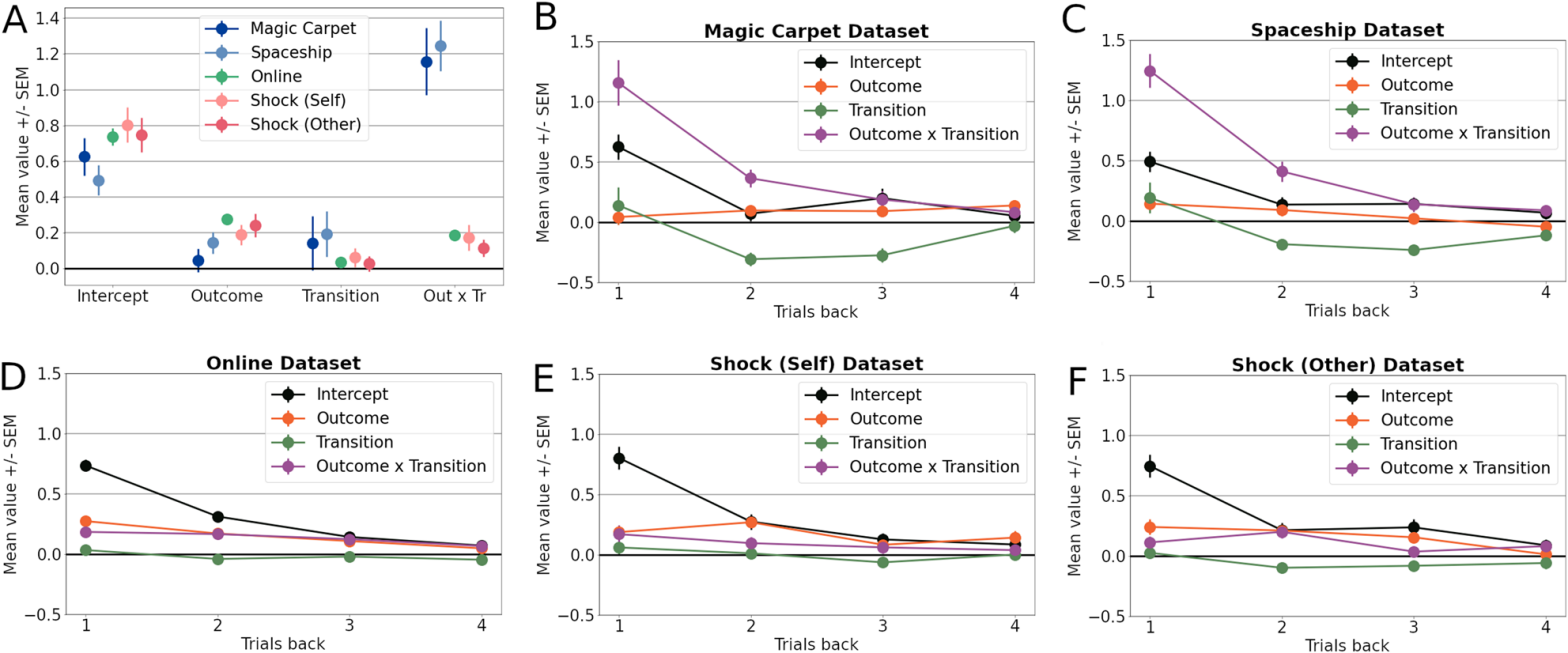
Logistic regression analyses. Regression analysis of first-stage actions based on the previous four trials. (**A**) Regression coefficients for the intercept, main effects of outcome and transition, and outcome-transition interaction only on the preceding trial across the different datasets. (**B**–**F**) Each subplot shows the coefficients for one dataset on all four preceding trials.

By inspecting the regression coefficients resulting from the logistic regression analyses, considerable differences in participant behaviour were observed across the datasets (Fig. 3). Most notably, behaviour in the Magic Carpet and Spaceship paradigms showed a greater outcome-transition interaction, a proxy of model-based inference. They additionally showed larger main effects of transition: small positive coefficients for the previous trial, with larger negative coefficients for behaviour two and three trials back. This indicates that common transitions lead participants to actively switch to the other initial-stage action independent of trial outcomes, potentially indicating information-seeking behaviour^6^. These effects were largely absent in the remaining datasets. Main effects of outcome were relatively small in the Magic Carpet and Spaceship tasks, while the intercept terms were comparable across all included datasets.

### Comparing hybrid reinforcement learning and active inference

**Figure 4:**
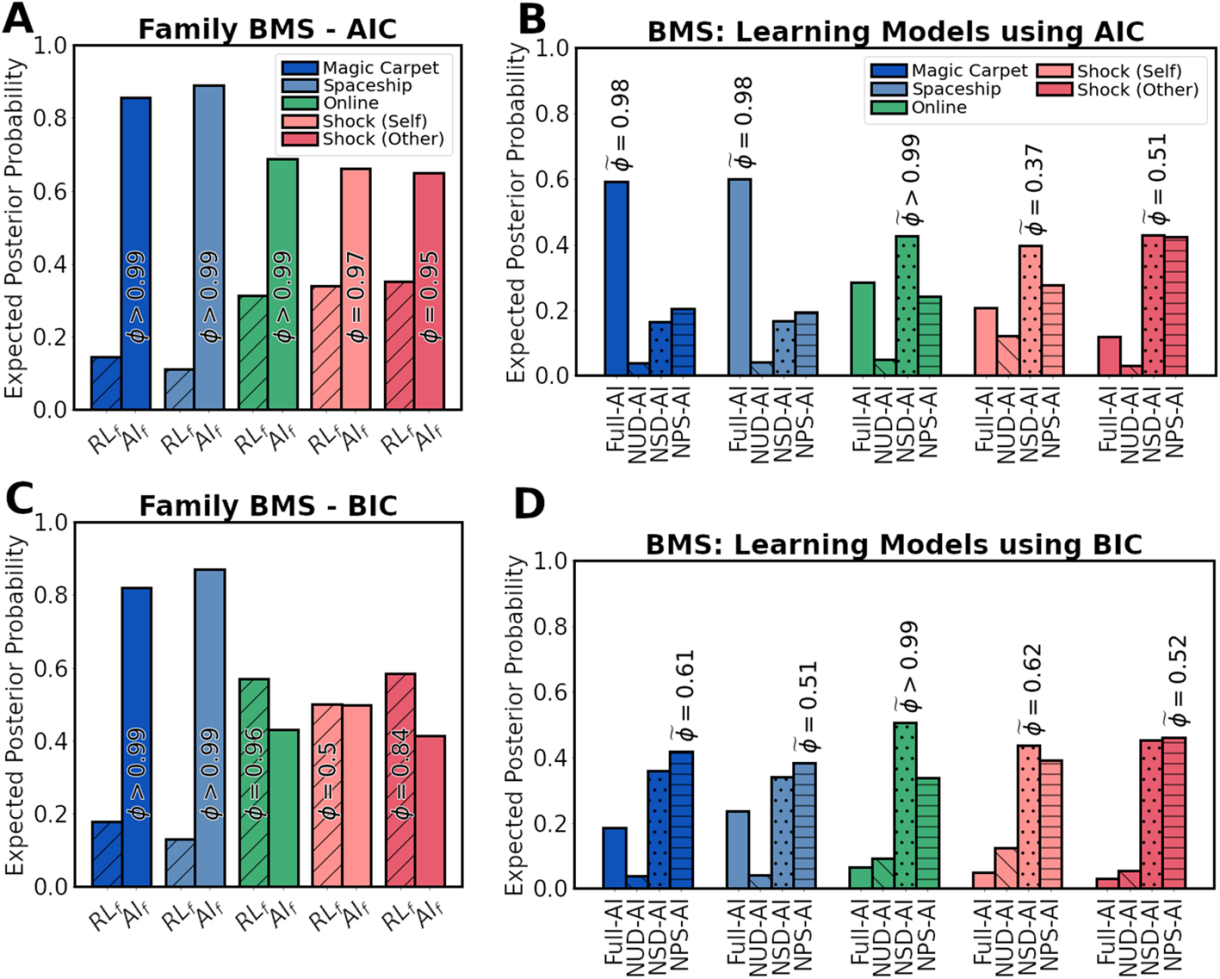
Bayesian model comparison. (**A**,**C**) Expected posterior probabilities resulting from the Bayesian Model Comparison between the $RL_f$ family containing the hybrid-reinforcement learning model and the $AI_f$ family, containing the active inference models with varying learning dynamics (see Fig. 2) using AIC (**A**) and BIC (**C**). $\phi$ indicates the exceedance probability in favour of the best-scoring family. (**B**,**D**) Expected posterior probabilities for the different learning models contained within the $AI_f$ family. ${\widetilde{\phi }}$ indicates the protected exceedance probability of the best-scoring model.

The hybrid reinforcement learning (Hybrid-RL) model and the active inference model were compared in their ability to explain participant choice data via Bayesian model selection. To do so independently of the variations of the probabilistic learning model displayed in Fig. 2, these were clustered into a family and compared against a family containing only the Hybrid-RL model. The active inference family was found to describe single-trial participant behaviour better for the Magic Carpet and Spaceship datasets (both exceedance probabilities $\phi >0.99$), with consistent results between AIC (expected posterior probabilities of $\langle r_{AI} \rangle = [0.86, 0.89]$ respectively) and BIC ($\langle r_{AI} \rangle = [0.82, 0.87]$ respectively) (Fig. 4A,C). For the other datasets, the metrics did not agree on the best performing family. Using AIC, the Online and Shock datasets slightly favoured Hybrid-RL ($\langle r_{RL} \rangle = [0.57, 0.50, 0.59]$), while the active inference family scored better using BIC ($\langle r_{AI} \rangle = [0.68, 0.66, 0.65]$).

The different learning models were subsequently compared against one another (Fig. 4B,D). In multiple cases, there was ambiguity about the best scoring model. For the Magic Carpet and Spaceship datasets, only AIC provided strong evidence for a model: the “Full”-variant with both belief decay and surprise-based learning (protected exceedance probabilities ${\widetilde{\phi }}=[0.98, 0.98]$ respectively). Using BIC, model scores were distributed across the Full, NSD, and NPS model variants. For the Online and Shock datasets, the simpler ’NSD’-model which omits decay of concentration parameters for the sampled action tended to score highest. However, this was only clearly the case for the Online dataset, with ${\widetilde{\phi }}>0.99$ using both AIC and BIC. Expected posterior probabilities were quite widely distributed over the Full, NSD, and NPS variants. This was also found for the Shock dataset, indicating that the best fitting learning model differed across subjects. As the Online dataset contained more subjects, exceedance probabilities were still high due to greater confidence a majority of subjects used the NSD variant. The NUD variant, omitting decay of concentration parameters of unsampled actions, scored poorly across all datasets and metrics. This indicates the importance for a decay of concentration parameters of beliefs about unsampled actions, likely to appropriately capture the behavioural flexibility observed in participants. Overall, a dissociation between learning models was incomplete.

#### Simulation analysis

**Figure 5:**
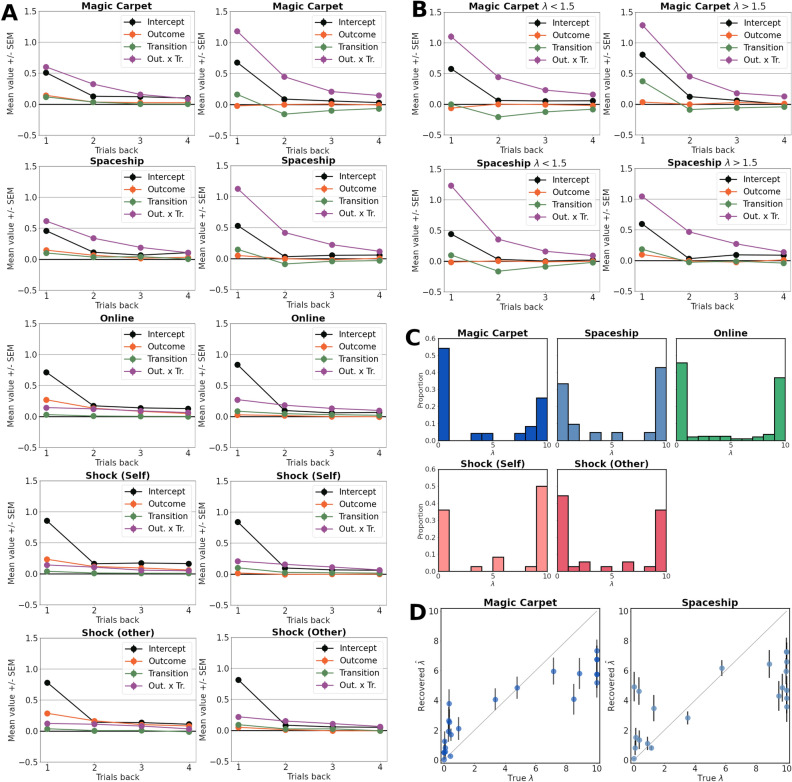
Simulation analyses. (**A**) The Hybrid-RL (Left) and active inference (Right) models were used to simulate behaviour on the two-step task using the maximum-likelihood parameters for each participant. The resulting data were subjected to the same logistic regression analyses as the participant data of Fig. 3. (**B**) Data was simulated for participants with low (Left) and high (Right) values of $\lambda$ separately. The coefficients resulting from the logistic regression are plotted per group. (**C**) The recovered values of the $\lambda$-parameter per dataset, displaying highly bimodal distributions. (**D**) A comparison of true but unknown $\lambda$-parameter values and the recovered values. Each datapoint depicts one participant.

To gain insight into the extent to which the models capture participant behaviour, we simulated model behaviour on the two-step task. The maximum likelihood parameter estimates were used and the resulting synthetic datasets were submitted to the logistic regression analyses (Fig. 5A). The Full model was used for the Magic Carpet and Spaceship datasets, while the NSD variant was chosen for the Online and Shock datasets. On the Magic Carpet and Spaceship datasets, the active inference model appears to better capture the strong interaction between outcome and transition type, while it is underestimated by the Hybrid-RL model. The active inference model displays minor main effects of transition, although not to the extent that they are present in the participant data, while being absent in the Hybrid-RL model. For the Online and Shock datasets, the smaller interaction terms and close-to-zero interaction terms are reproduced by both models. The main-effects of outcome are reproduced by the Hybrid-RL model, but not by active inference.

### Correlation analyses

To follow up on the differences between the model families, we inspected potential contributions to differences in model fit. Specifically, as regression analyses suggest that behavioural datasets which are better explained by active inference show both greater model-based inference as well as potential directed exploration, we investigated which of these correlated with relative model fit. We additionally compute correlations between the $\lambda$-parameter and the main-effects of transition to understand whether this averaged-based metric indeed tends to be greater for subjects sensitive to information-gain as identified by a low $\lambda$-parameter.

The correlation analyses between single-subject relative model fits and the interaction term between outcome and transition type (as a proxy for model-based inference) were positive and larger for the Magic Carpet (Pearson’s $r=0.71$, $p<0.001$) and the Spaceship ($r=0.63$, $p=0.002$) datasets (Table 1). For the Online and Shock (Other, Self) datasets, correlation coefficients were lower ($r=[0.19, 0.18, 0.08]$, $p=[0.009, 0.29, 0.63]$ respectively). As the $\lambda$-parameter was not normally distributed, we adopt two common approaches. First, we offset the data such that the lowest value was 1, allowing for a log-transformation of the data and computing Pearson’s *r* ($r_p$). Second, we used the data without transformation to compute the non-parametric, rank-based correlation measure Spearman’s *r* ($r_s$). Repeating the aforementioned analyses for the $\lambda$-parameter and relative model fit, a similar contrast between datasets appeared. Modest correlations were observed in the Magic Carpet ($r_p=-0.35, p=0.09$, $r_s=-0.14, p=0.51$) and Spaceship ($r_p=-0.45, p=0.04$, $r_s=-0.40, p=0.07$) datasets. Meanwhile, the correlations were smaller for the Online and Shock datasets ($r_p=[0.10, 0.18, 0.08]$, $p=[0.16, 0.29, 0.66]$ and $r_s=[0.09, 0.11, -0.10]$, $p=[0.26, 0.53, 0.56]$, respectively). The correlations between the $\lambda$-parameter and the transition-coefficients were again somewhat larger for the Magic Carpet ($r_p=0.29, p=0.16$, $r_s=0.26, p=0.23$) and Spaceship ($r_p=0.55, p=0.01$, $r_s=0.34, p=0.13$) tasks, compared to the Online and Shock datasets ($r_p=[0.14, -0.08, -0.07]$, $p=[0.06, 0.63, 0.70]$ and $r_s=[0.09, 0.07, -0.17]$, $p=[0.23, 0.70, 0.33]$, respectively).

### Evidence for directed exploration

Next, the role of the $\lambda$-parameter was further investigated. The distribution of parameter values resulting from the maximum-likelihood procedure is displayed in Fig. 5C. In every dataset, a bimodal distribution was found, with a majority of participants having a $\lambda$-parameter of smaller than one or close to the upper bound of ten (Fig. 5C). To analyse the effect of the $\lambda$-parameter on the main-effect of transition in the regression analysis directly, participants were stratified into two groups: a low $\lambda$ group ($\lambda <1.5$) and a high $\lambda$ group ($\lambda >1.5$). The associated model parameters were subsequently used to simulate group-specific behaviour on the two-step task. The low $\lambda$ group displayed moderately stronger transition effects than the high $\lambda$ group (Fig. 5B). Nevertheless, even this low $\lambda$ group did not exhibit transition effects to the extent that these were observed in behavioural participant data.

**Table 1:** Correlation analyses.

Paradigm	corr($\beta _{o \times \tau }^{t' -1}$, $\Delta {\hat{L}}$), (*p*)	corr($\lambda$, $\Delta {\hat{L}}$), (*p*)	corr($\lambda$, $\beta _\tau ^{t'-2,3}$), (*p*)
Magic Carpet	**0.71** (< 0.001)	− 0.35 (0.09)/− 0.14 (0.51)	0.29 (0.16)/0.26 (0.23)
Spaceship	**0.63** (0.002)	$-$ **0**.**45** (0.04)/− 0.40 (0.07)	**0.55** (0.01)/0.34 (0.13)
Online	**0.19** (0.009)	0.10 (0.16)/0.09 (0.26)	0.14 (0.06)/0.09 (0.23)
Shock (Self)	0.18 (0.29)	0.18 (0.29)/0.11 (0.53)	− 0.08 (0.63)/0.07 (0.70)
Shock (Other)	0.08 (0.63)	0.08 (0.66)/− 0.10 (0.56)	− 0.07 (0.70)/− 0.17 (0.33)

Correlation coefficients and uncorrected, associated *p* values. Due to $\lambda$ not being normally distributed, both Pearson’s r using log-transformed data (left) and Spearman’s r (right) using untransformed data are presented for its analyses. $\beta _{o \times \tau }^{t' -1}$: regression beta-coefficient for the interaction between outcome and transition type for the preceding trial, $\Delta {\hat{L}}$: difference in values of maximized log likelihood function for the Hybrid-RL and best fitting active inference model, $\beta _\tau ^{t'-2,3}$: beta-coefficient encoding the main effect of transition type at the second and third previous trials.

Significant values are in [bold].

In an exploratory analysis, we compared whether task performance in terms of average obtained reward differed between the low and high $\lambda$ groups. A Welch’s t-test was used due to unequal variances and sample sizes for some datasets. The high $\lambda$ group obtained slightly more reward on most datasets ([+ 11.9%, + 4.0%, + 1.3%, − 0.5%, + 3.3%] for the Magic Carpet, Spaceship, Online, Shock (Self) and Shock (Other) datasets respectively), with uncorrected $p<0.05$ for the Magic Carpet dataset ($t (p) = [-2.28 (0.03), -0.49 (0.47), -1.04 (0.29), 0.12 (0.91), -0.94 (0.35)]$).

Finally, we verified that the $\lambda$-parameter could be accurately recovered given the constellation of model parameters as identified in the participant sample. Synthetic choice data was repeatedly generated using the set of parameters of every subject, which subsequently underwent parameter fitting. The results of this recovery analysis for the $\lambda$-parameter is displayed in Fig. 5D. Correlations between true and recovered parameter values were high for both the Magic Carpet ($r_p=0.85, p<0.0001, r_s=0.90, p<0.0001$) and Spaceship tasks ($r_p=0.74, p=0.0001, r_s=0.60, p=0.004$). Parameter values were often recovered accurately for low values of $\lambda$, although especially in the Spaceship dataset this was not successful for all participants. Many of the high $\lambda$-parameter values were underestimated, which likely stems from increases in $\lambda$ having diminished effects. As $\lambda$ increases past values of approximately 3, the expected free energy is already heavily biased towards preference maximization. For example, due to the linear scaling an increase in $\lambda$ from 0.1 to 0.5 is comparable in size to an increase from 2 to 10. The large upper bound on $\lambda$ of 10 was to ensure a pure preference-realization strategy could be captured by the model-fitting procedure and not due to the idea that variations in large $\lambda$ values would meaningfully change behaviour. Overall, for most participants, the $\lambda$-parameter could be recovered successfully, although with considerably noise and inaccuracy for multiple subjects.

## Discussion

In this paper, we collected and analysed datasets of human participants performing the two-step task and compared the explanatory power of the active inference framework to that of the standard hybrid reinforcement learning (Hybrid-RL) model. Logistic regression analyses revealed marked differences in initial-stage choice behaviour of participants between datasets, with two datasets exhibiting markedly greater model-based inference. Bayesian model comparison indicated that only these two datasets were significantly better described by active inference. The degree to which participants used model-based inference was found to strongly correlate with the relative performance of the models, while the exploration-exploitation parameter $\lambda$ showed only a small to moderate relationship. Model simulations indicated that the probabilistic learning underlying active inference likely contributed to the better model fits and that exploration behaviour of subjects was better captured by active inference, albeit only partially. This was the case even when analysing only those subjects who were classified as most strongly pursuing directed exploration.

The Magic Carpet and Spaceship datasets featured comparatively small effects of outcome yet stronger effects of both transition type and the interaction of outcome and transition type. This replicates the results reported by da Silva and Hare^6^, suggesting a greater degree of model-based inference in these datasets compared to the Online and Shock tasks. The Shock study^37^ provided thorough instructions to participants, as did the Online study^36^. However, neither provided detailed explanations for all aspects of the task, such as transition type. da Silva and Hare^6^ argued that providing intuitive reasons for task dynamics aided participants in adopting a more accurate model of the task. This is plausible as task knowledge is known to be able to influence the task model^50^, with direct comparisons having been performed for the two-step task specifically^51^. Although the current work does not aim to explain why behaviour differed between tasks, we recover a similar behavioural discrepancy.

By partitioning model space, we were able to compare the Hybrid-RL model to active inference independent of the specific learning model. The discrepancy between datasets also persisted on this level, as we showed strong evidence in favour of active inference for the Magic Carpet and Spaceship datasets, yet there was no consensus between AIC and BIC for the Shock and Online datasets. As BIC penalizes models for their complexity more than AIC does, a lack of consensus implies that the additional parameters in the active inference models did not sufficiently increase the fit of the model. As such, additional caution is warranted when inspecting the comparison of the individual active inference models for the Shock and Online datasets.

Overall, we were unable to convincingly dissociate between learning models, indicated by the lack of consensus between metrics. Nevertheless, on the Magic Carpet and Spaceship tasks, the more complex, full model scored well using AIC, combining both decay and surprise-based learning for chosen actions. A trend was observed where a simpler model without decay of concentration parameters for sampled actions scored best for the Online and Shock datasets. This model only uses surprise-based learning to update beliefs about outcomes of chosen actions, although a model with only decay and without surprise-based learning scored similarly. These results suggest that learning dynamics are the result of only decay or surprise-based learning, but not both. The high protected exceedance probabilities but low posterior probabilities for the Online dataset likely result from the large amount of participants. This combination suggests a very high probability that a model without decay is most prevalent in the sample, yet behaviour of many participants is best described by other models. A consistent finding, however, is the very low scoring of the model without decay of beliefs about unsampled actions. This may highlight an important distinction between the learning rule based on probability distributions and the Hybrid-RL model. Using probabilistic learning, sampling an action increases the concentration parameters of a belief distribution, often equated to increases in confidence^52^. In absence of a decay or forgetting mechanism, these higher concentration parameters impede flexible behaviour when an action is revisited at a later time and appears rewarding. Thus, the poor scores of this model imply the use of such a decay mechanism by participants in the Magic Carpet and Spaceship tasks. Due to the lower scoring of active inference in the remaining datasets, it could alternatively indicate that rather than using belief distributions, participant behaviour on the Online and Shock datasets is best described as learning point estimates (such as Q-values in reinforcement learning), which intrinsically allow for continued flexible behaviour. Nevertheless, forgetting mechanisms are commonplace in cognitive and neuroscience^46,53,54^ and have been previously shown to improve model fits on the two-step task^55,56^.

To gain insight into what drives the relative model scores, we simulated behaviour using the Hybrid-RL and best fitting active inference models. The strong positive interactions between outcome and transition type observed with the Magic Carpet and Spaceship tasks were reproduced by the active inference model, but not the Hybrid-RL model. This interaction has generally been interpreted to show model-based inference^3^, but using the current analyses by Miller et al.^38^ shows the progression of this term over trials and thereby reveals further information about the learning dynamics. Besides the strength of the interaction, its relative and diminished influence on preceding trials shows the sensitivity of beliefs to the history of observed outcomes. As such, it is mainly interpreted as a reflection of underlying learning dynamics rather than a function of action selection. The ability to capture this interaction term accurately thus further shows promise for the role of probability distributions to model learning on the two-step task, displaying a better description of behaviour than the Q-value learning strategy of the Hybrid-RL model. This finding fits with the general idea underlying the Bayesian brain hypothesis^23^. However, this advantage does not extend to the Online and Shock datasets, as the weaker interaction-terms are reproduced by both models.

The main effects of outcome have been interpreted as a proxy of model-free inference^3^. These effects, which are especially prevalent on the Online and Shock datasets, are not captured by the active inference model as the underlying learning rule we used is purely model-based. This discrepancy between human behaviour and active inference simulations likely contributes to the lower active inference scores on the model comparison analyses for these two datasets. Although active inference often receives a (generative) model-based treatment in the literature^21,57,58^ and is therefore the focus of the current work, the framework is not incompatible with multi-system theories (such as the co-existence of separate model-free and model-based inference under the Hybrid-RL model). For example, active inference as implemented here could be used in conjunction with algorithms more similar to the model-free part of the Hybrid-RL model, which may be implemented as a form of habitual behaviour^19^. This could form the basis of interesting future extensions and may lead to a model better able to account for the distinct behaviour observed across datasets. Nevertheless, such an approach may only yield marginal improvements for the datasets investigated here. First, behaviour in the Online and Shock datasets does not reveal strong main effects of transition type and are thus unlikely to benefit from modelling directed exploration via expected free energy. Second, main effects of outcome are minor on the Magic Carpet and Spaceship tasks, which are thus unlikely to feature significant model-free behavioural components. Taken together, the simulation analyses thus far appear to confirm the model comparison analyses which indicated only the Magic Carpet and Spaceship datasets to be better described by active inference.

A critical component of active inference is its formulation of the exploration-exploitation trade-off. The studies by da Silva and Hare^6^ included post-hoc reports of subjects, which included descriptions of intentionally visiting a specific final-stage state multiple times, before actively aiming for the other state. These reports are congruent with the observed transition effects and the authors proposed this may indicate directed exploration behaviour. The main effects of transition type observed on the Magic Carpet and Spaceship datasets are considerably weaker in the active inference simulations. For the active inference model to provide a behavioural description that is incompatible with a pure reward driven strategy, sufficient weight needs to be assigned to the information gathering term. This was found for approximately half of all subjects, suggesting that sensitivity to information gain was common. Although necessary, it is not a sufficient condition to produce significant effects of transition-type. For example, small learning rates, as well as small decay rates, diminish the effect of new observations on beliefs and thereby also decrease the information gain term of the expected free energy. Thus, noticeable transition-type effects also require observations to be able to significantly impact beliefs under current model assumptions. By performing the simulation analyses for subjects assigned a low or high $\lambda$-parameter separately, we show that this parameter does contribute to the main-effects of transition when using participant parameters, although the stratification of the datasets leads to small sample sizes. The correlation analyses support this, as participants with greater transition effects tend to be assigned lower $\lambda$ values. In addition, these lower parameter values appear associated with relatively better model fits for active inference. However, the correlations are small-to-moderate in strength and even the simulations for the low $\lambda$-parameter group still do not fully capture the transition effects seen in the behavioural data. It is important to note that the two-step task was not specifically designed to disambiguate directed exploration strategies. Participants in the low $\lambda$ group did not obtain more reward, probably due to the little influence agents have on the obtained rewards as shown by Kool et al.^36^. Ultimately, as per the exploration-exploitation trade-off, exploration is only worthwhile if sacrificed short-term reward can be recouped by utilizing obtained information via different action selection strategies. In sum, active inference as implemented here likely does not provide a full description of observed exploration behaviour, although surrogate tasks should be considered.

Alternative specifications for an information-bonus have been considered by previous research. Compared to computing the expected Kullback-Leibler divergence, simpler accounts include an ’all-or-nothing’ bonus, in which the least-explored option receives a fixed information bonus^59^. Others have modelled directed exploration by scaling the information-bonus linearly by using a count of the number of times an option has been selected^15^. More sophisticated strategies are time-horizon dependent^60^, a further possible extension to the current active inference implementation already discussed in the literature^61^. Another possibility altogether is the existence of distinct exploration and exploitation states, rather than a weighted trade-off that is computed on a single-trial basis^11^.

An important issue for studying exploration is the coupling of reward and information. In many paradigms, including the two-step task, participants receive only outcome information about the selected action. To the extent that participants act to obtain rewards, more rewarding actions naturally tend to be sampled more often, thus correlating how much information participants have about an option and how rewarding it is^59^. Researchers have attempted to manipulate the value of either strategy using ideas such as forced sampling^15^ and periodic introductions of novel options^62^. Additionally, Horvath et al.^63^ modeled a bandit-task using an approach similar to active inference, but withheld reward-information on a subset of trials. This allowed for a demonstration of human sensitivity to an information-bonus computed as the expected Kullback-Leibler divergence. As the two-step task was designed for the disambiguation of model-free and model-based inference, the resulting lack of decoupling between reward and information may have interfered with identifying a directed exploration strategy due to its correlation with a reward-maximization strategy. The level of noise in the parameter recovery supports this interpretation.

Some considerations of the current work deserve mention. First, due to interactions between learning and action selection processes, it is possible active inference would explain exploration behaviour better when combined with a different learning algorithm than ours. This may be the case directly with respect to the employed learning rule, for example by using a hierarchical model that estimates environmental volatility. It could, however, also be a function of the (misconstrued) task model, on which directed exploration relies to predict information gain. Secondly, the current study implements active inference by specifying expected free energy as the loss function for action selection. Over time, the scope of active inference in the literature has been extended to also include learning and inference schemes, often based on message-passing implementations^64^. Although we focused on final-stage outcomes to stay close to the two-step task literature, the exploration of more expansive applications of the framework may be considered in the future, including potential information-gain incentives for inference about the transition structure. Future research might also extend its scope beyond directed exploration incentives to explore dynamic habitual control via learned, rather than static, habits, which might allow for an active inference-based analogue to model-free inference. Moreover, the Hybrid-RL model learns point estimates and active inference uses probability distributions, preventing direct comparisons of action selection strategies on this task. This complicates the interpretation of relative model fits as these may result from differences in either learning or action selection. Nevertheless, we aimed to mediate this issue by using additional logistic regression analyses instead of relying only on model comparisons. Overall, the aim to investigate the applicability of active inference in describing data not specifically recorded for this purpose using diverse datasets did complicate drawing certain conclusions. As such, the heterogeneity of the datasets is difficult to fully account in part due to considerable differences in sample sizes. The analyses presented here should thus be regarded as a proof-of-principle to steer future work testing the empirical validation of active inference.

To conclude, we replicated results by da Silva and Hare^6^ and extended on behavioural discrepancies between datasets of the two-step task. Participants in the Magic Carpet and Spaceship datasets not only appeared to perform more model-based inference, but also showed more volatile learning dynamics and greater transition effects, which indicate the use of directed exploration strategies. These two datasets were better described by an active inference model than a Hybrid-RL model, while the models scored similarly for the Online and Shock datasets. For the Magic Carpet and Spaceship datasets, the use of a learning model based on probability distributions appeared to contribute to the better model fits and captured behaviour better than the Hybrid-RL model. For such a model, a decay mechanism for beliefs about unsampled options was found to be important. Model parameters indicated that approximately half of all subjects were sensitive to information gain of actions. However, active inference was only partly able to capture the observed transition effects, and thus likely did not provide a full account of exploration behaviour.

## Data Availability

The datasets analysed during the current study were previously made available by Kool et al.^36^ (github.com/wkool/tradeoffs), Lockwood et al.^37^ (osf.io/3stp9/files), and da Silva and Hare^6^ (github.com/carolfs/muddled_models).

## Code availability

The modeling code used in this work is available at github.com/SamGijsen/AI2step.
